# Factors associated with nurses’ perceptions, their communication skills and the quality of clinical handover in the Hong Kong context

**DOI:** 10.1186/s12912-021-00624-0

**Published:** 2021-06-11

**Authors:** Jack Pun

**Affiliations:** Department of English, City University of Hong Kong, 83, Tat Chee Avenue, Kowloon, Hong Kong SAR, China

**Keywords:** Nursing handover, Communication, Training, Evidence-based practice, Perceptions

## Abstract

**Background:**

Clinical handover is a pivotal, high-risk communicative event because it involves the transfer of responsibility and accountability for patients and their care. Nurses’ perceptions and their communication skills inevitably impact on their ability of clinical handover. Limited studies have explored nurses’ handover practice in the Hong Kong context. This study aimed to identify factors associated with and specific impact paths between the quality, communication skills and nurses’ perceptions on clinical handover.

**Methods:**

A questionnaire survey was conducted immediately after the nurses’ training in effective handover communication. A convenience sample of 206 bilingual nursing staff from a local hospital in Hong Kong participated in this paper-and-pencil survey adopted from the Nurses Handover Perceptions Questionnaire survey.

**Results:**

The path analysis revealed that except the opportunity to ask questions and high perceptions of the ISBAR communication protocol, other factors were significantly correlated with improved quality of handover. In addition, nurses who had updated information were likely to ask more questions and obtain a better understanding of the patient care plan during handover.

**Conclusions:**

The quality of nursing handover depended on the degree of nurses’ grasp of the patient care plan. The ISBAR communication protocol was considered helping nurses to improve their communication skills with other colleagues and indirectly enhance patient’s safety. However, although ISBAR facilitated nurses to structure clearer handover communication, it was not the most important predictive factor for determining handover quality.

**Supplementary Information:**

The online version contains supplementary material available at 10.1186/s12912-021-00624-0.

## Introduction

Handoff or handover was a fundamental routine clinical practice for the effective transfer of patient care plan between health professionals [1]. When a patient was ‘handed over’ by an outgoing nurse to an incoming nurse between shifts, communication about the patient’s condition was important to ensure appropriate continuity of care. Failure to understand a patient’s condition, acquire updated information about the patient, or ask questions to clarify information during handover would put the patient at risk. In particular, dire events, inadequate care and delayed treatment might be caused by the nurses’ failure to share all relevant clinical information of the patient accurately and timely.

## Background

Nursing handover represents one of the most important transition points for responsibility and accountability in patient care among nurses [2, 3] as it often consists of a range of vital information, the patient’s diagnosis and treatment plan, for instance. Whilst studies have reported that many handover practices are not structured, which suggests that there was a lack of meticulousness and efficiency when sharing important clinical information among all nursing staff. On top of the complexity of handover communication in today’s dynamic clinical management, there has been a surge in using communication protocols such as the I-PASS mnemonic or ISBAR (Introduction, Situation, Background, Assessment, Recommendation) [4, 5], currently known as ISBARQ (Introduction, Situation, Background, Assessment, Recommendation and Question and answer), in the hospitals to guide nurses to deliver information regarding patients’ conditions in a structured and effective manner [6–8], as well as promoting seamless exchange and complete understanding of a patient’s condition and care plan [9].

Handover communication protocols are designed to help nursing staff to structure their handover communication and present patients’ information in a logical and coherent manner, with the aim of reducing the possibility of miscommunication or misunderstanding between the nursing professionals. Research demonstrated that a clear communication structure for nursing handover was highly beneficial to ensure that clinicians cover each important area of information [10] and provide opportunities for clarification to both the giver and the receiver of patients’ responsibility [11–13]. Gardiner et al. [14] found that technical errors during handovers and high-risk events following handovers can be reduced if there is an appropriate use of a structured, standardised clinical tool for handovers, as they provided an effective format for the delivery of comprehensive and accurate medical information, with fewer omissions [15]. Moreover, a handover communication structure allows the facilitation of interdisciplinary clinical teams and hospital staff at different levels of hierarchy to work together [16, 17].

The ISBARQ protocol, which is one of the recognised handover communication frameworks, has gained popularity worldwide in assisting health professionals with structuring handover communication in an organizational format. Specifically, in the context of Hong Kong, ISBARQ has been the most popular tool used by local frontline staff, according to the Hospital Authority [18]. It is also the preferred framework used in the participating hospital in the current study. Meanwhile, despite the encouragement of using standardised communication framework from the Hospital Authority, variations in the patterns of nursing handover communication and content have been identified in the hospitals in Hong Kong [19], the study site in this study included. Some nurses were able to structure their information in a logical organised sequence, whereas others tended to follow a narrative and descriptive approach. Such discrepancy in the style of conveying information at handover, however, was shown to cause adverse effects on the patient. One of the consequences was a set of confusing, incomplete or ambiguous set of handover content as a result of the lack of consistent and logical structuring in handover communication as reported by Eggins et al [20].

These informational problems were often compounded by the fact that nurses could rarely interact during handovers given the hectic clinical setting. Except for time constraints, nurses commonly reported that speaking up to share their opinions and concerns during handover is challenging due to a strong sense of hierarchy [21, 22]. Such findings hence infer that even there remained information unclear during handovers, nurses seldom had the opportunity, or would rarely proactively ask for clarification [23, 24].

Furthermore, some researchers have reported possible limitations of standardised handover tools [25–27], in which one example being the use of a standardised checklist did not necessarily lead to a high level of consistency during handovers even for experienced nurses [20]. Additionally, several studies about the efficacy of the implementation of ISBAR on nurses’ handover performance also showed that despite a higher-frequency query of ambiguous information, errors still occurred during handover, which in turn led to a lower self-perceived quality of handover practices [28, 29]. Eggins and Slade’s [13] paper investigating the informational and interactional aspects of clinical handover revealed that the narrow understanding of communication significantly impeded nurses’ communication capabilities during handovers at clinical settings where the ISBAR tool was introduced. The previous findings imply that the quality of handover does not rely only on the application of a standardised handover tool, nurses’ communication skills and their understanding of ISBAR might also be potential factors that predict nurses’ quality of handover.

As suggested by Anderson et al. [30], there was an absence of studies that fully explain how to optimise handovers. Whilst previous literature has overall pointed that the links between how nurses perceive the ISBAR protocol, the type and degree of information they obtain during handover, and their understanding of handover communication to be investigated. The purposes of the current study were therefore to identify the factors associated to nurses’ perceived quality of handover, and to hypothesise a model to illustrate specific impact paths on how such factors may be correlated.

## Methods

### Aims

This study aimed to identify factors and specific impact paths among nurses’ perceptions, communication skills and quality of handover.

### Participants and data collection

A post-evaluation of nurses’ handover using a questionnaire survey was conducted immediately after a training workshop on effective handover communication. The training was a 3-h program that integrated re-enacted videos of handover interactions with role-play simulations, with ISBAR utilised as a checklist. A convenience sample of 206 bilingual nursing staff from a local hospital in Hong Kong participated in this paper-and-pencil survey. All 206 nurses received prior basic handover training yet were not specifically trained on using ISBAR or other recognised handover frameworks; they all worked in a Cantonese-English bilingual medical setting in Hong Kong, and participated in the study voluntarily.

### Instrument

To measure the nurses’ perceptions of their communication skills and the quality of handover, a validated 22-item questionnaire named the Nurses Handover Perceptions Questionnaire (NHPQ) was adopted [11] (See the supplementary material). The NHPQ items were originally adapted from Klim et al. [31] and Street et al. [32], which examined nurses’ perceptions of their current practices and essential components of effective shift-to-shift nursing handovers. This instrument has now been psychometrically validated and used in Hong Kong to evaluate nurses’ perceptions and practices surrounding handovers [17]. Each of the 22 survey items were evaluated by a panel discussion among 10 head nurses and ward mangers to check on the clarity of the wording. Cronbach’s alpha was used to assess the internal reliability of the questionnaire items. The 10 head nurses and ward managers partook in the test-retest assessment twice, with 1 week apart. A high degree of reliability of Cronbach’s alpha 0.99, with a 95% confidence interval exceeding 0.7 was yielded among the responses.

To tailor to the current study, the NHPQ has been revised as a 21-item questionnaire survey, comprising of a series of statements about nurses’ overall perceptions of handovers and their experiences in clinical handover practice. More specifically, the statements focused on nurses’ views of the presentation, organisation, comprehension and dissemination of patient information, perceptions of ISBAR. Overall, the NHPQ covered seven characteristic features of effective clinical handovers: 1) providing adequate information about patients (questions 1,3,4,11,16–18); 2) organising information clearly; using of handover check sheets/charts (questions 2,5–7); 3) comprehension of receiving handovers (questions 8,15); 4) communicating skills effectively (questions 9,10); 5) seeking further information (question 12); 6) asking questions and resolving concerns (questions 13,14); 7) creating a clear patient plan; use of ISBAR and perception of ISBAR (questions 19–21). Participants were instructed to respond on a 4-point Likert scale, where 1 = strongly disagree, 4 = strongly agree.

Among the items, six were selected as five scales for the measurement, and some items were reversely phrased to optimise the expression of meaning for the present study. The quality of handover focused on whether the handover information presented by nurses was systematic and organized. To measure the features of handover information received by nurses after the training, an item was included for them to determine if the handover they received was up to date. Another item regarding the clear understanding of the plan (diagnosis, treatment, discharge, etc.) for the patient(s) was included to explore the effect of post-training. In a similar vein, an item asking if the participants had opportunities to ask questions that they did not understand during the handover was also selected. Besides, two items related to the perception of ISBAR (a. I believe using ISBAR will help me to improve communication skills with my co-workers; b. I believe using ISBAR will increase patient quality and safety care) were combined into a single item on the revised measure, and showed acceptable evidence of internal consistency (Cronbach’s α = .92).

### Ethical consideration

Ethical approval was obtained from the Research Ethics Committee of the participating hospital (Ref. no. 2017–07). The participants received an explanation about the objectives of the research project, as well as understood their right to withdraw at any time and an assurance of confidentiality. Written informed consent was received from all the participants during each phase of the project. All methods were carried out in accordance with relevant guidelines and regulations.

### Data analysis

**Fig. 1:**
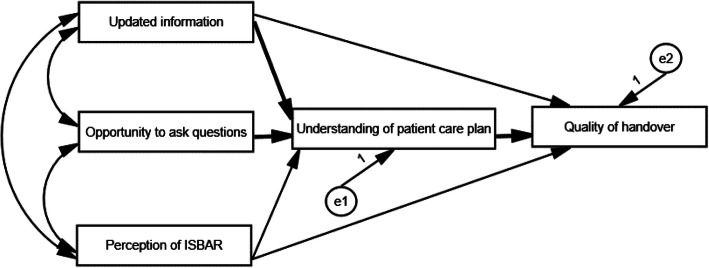
The hypothesised model

The demographic information of the participants was described using descriptive statistics, and correlations between the variables were evaluated. Normality test was performed using the SPSS software, showing that the data was normally distributed. In this research, there were less than 3% of the missing data on the survey items of sex and age, respectively, from the participants’ responses. The assumptions of path analysis were met and path analysis was performed using the AMOS 21.0 program; Fig. 1 presented the relationships between the variables of the hypothesised model. In general, because the chi-square statistic (χ^2^) was sensitive to sample size, other stable indices such as the root mean square error of approximation (RMSEA), comparative fit index (CFI) and Tucker–Lewis index (TLI) needed to be considered simultaneously. Therefore, all of the χ^2^, RMSEA, TLI and CFI were utilized to estimate the model fit. An RMSEA of .05 or less was considered as good, and values greater than 0.9 for the CFI and TLI also demonstrated a reasonably good model fit [33]. Maximum likelihood estimation with robust standard error was used to estimate the model’s parameters.

## Results

Table 1 presented the participants’ demographic information. Majorities of nursing staff were female (*n* = 186, 90.30%), had more than 6 years of work experience (*n* = 135, 65.6%), and aged between 30 and 39 years (*n* = 79, 38.30%). Of the 206 participants, 100 (48.50%) participants obtained a Bachelor’s degree, and 73 (35.50%) of them obtained a postgraduate qualification of Master’s degree or above.

**Table 1:** Descriptive statistics

Items		N	Percentage
Sex	Female	186	90.30%
	Male	14	6.80%
	Missing data	6	2.90%
Age	20–29 years	69	33.50%
	30–39 years	79	38.30%
	40–49 years	41	19.90%
	≥50 years	14	6.80%
	Missing data	3	1.50%
Education	Diploma	33	16.00%
	Bachelor’s	100	48.50%
	Master’s and above	73	35.50%
Work experience in current hospital	0–1 year	12	5.80%
	2–5 years	59	28.60%
	6–10 years	71	34.50%
	> 10 years	64	31.10%
Total		206	100%

Table 2 showed the bivariate correlations between the variables. Except for the opportunity to ask questions and perception of ISBAR, other variables were significantly correlated with the quality of handover. In addition, updated information was significantly correlated with the opportunity to ask questions and the understanding of the patient care plan.

**Table 2:** Bivariate correlations between the variables

	(a)	(b)	(c)	(d)	(e)
(a) Quality of handover	1				
(b) Updated information	.174*	1			
(c) Opportunity to ask questions	.133	.325**	1		
(d) Understanding of patient care plan	.242**	.388**	.380**	1	
(e) Perception of ISBAR	.051	.095	−.029	.011	1

Note: *p<.05; **p<.01

**Fig. 2:**
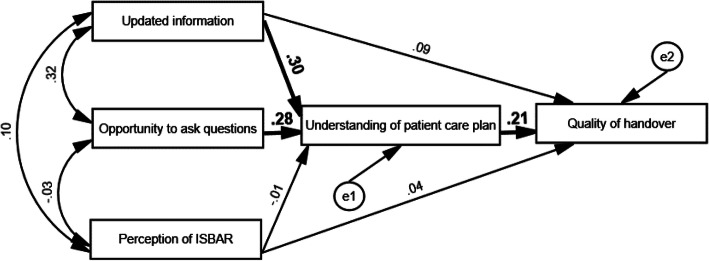
Hypothesised model with path coefficients

The indices of the hypothesised model indicated a very good model fit [χ^2^(1) = .193, *p* > .05, RMSEA = .000, TLI = 1.099, CFI = 1.000] (Fig. 2). As shown in Table 3, three of the hypothesised paths were significant. The quality of handover was directly correlated with the understanding of the patient care plan, which in turn was significantly correlated with updated information and the opportunity to ask questions. No significant path was found between the perception of ISBAR and other variables.

**Table 3:** Coefficient of the paths

Model path	Unstandardised coefficient (S.E.)	Standardised coefficient
Updated information → understanding of patient care plan	.304 (.067)***	.297
Opportunity to ask questions → understanding of patient care plan	.229 (.053)***	.283
Perception of ISBAR → understanding of patient care plan	−.005 (.034)	−.010
Understanding of patient care plan → quality of handover	.224 (.080)**	.206
Perception of ISBAR → quality of handover	.024 (.040)	.040
Updated information → quality of handover	.101 (.082)	.091

Note: **p<.01; ***p<.001

Figure 2 presented the path of the quality of clinical handover with path coefficients. Of the three preliminary factors, nurses who received updated information were prone to asking more questions. Additionally, updated information and opportunity to ask questions were found to significantly relate to the understanding of patient care plan, and were indirectly correlated to the quality of handover. Whilst our findings showed no significant correlation between the perception of ISBAR and the understanding of patient care plan, nor significant correlation between the perception of ISABR and the quality of handover.

## Discussion

Clinical nursing handover was a routine yet pivotal, high-risk communicative event in hospital. Nurses’ formal shift-end handovers occurred at least three times a day, excluding the in-between breaks or patient transfer. Studies highlighted insufficient and unstructured nursing handover as a big reason to avoidable critical incidents [5, 6, 8]. Effective and accurate communication between nurses during handover was therefore critical in ensuring safe and consistent quality of healthcare. Moreover, the World Health Organization had also listed improvement in handover communication as a part of the top patient safety solutions [34].

Meanwhile, variations in the patterns of communication at nursing handovers were commonly reported in the hospitals in Hong Kong [19]. A lack of consistent structure of communication for handover could result in misunderstandings where serious consequences could happen to the patients due the differences in information conveyance. The absence of interactions could further escalate the issues of an unclear handover practice. Without explicit query of information retainment or understanding, it was nearly impossible for the outgoing nurses to know whether the information has been successfully transferred to the incoming nurses.

Therefore, the ISBARQ protocol, which allows nurses to follow a narrative and descriptive approach to communicate during handover, has become the most popular tool that aids the facilitation of a focused, relevant and organised communication between nurses in Hong Kong [18]. The structure of ISBARQ provided a synopsis of the patient’s medical status; identified problems; listed assessment findings, intervention(s) and suggestions for the incoming nurses; and allowed the evaluation of patient care outcomes to enable safe and consistent nursing care [20]. Thus, the ISBARQ protocol ensured that information was effectively transferred at handover regardless of the clinical context or the number of nursing staff [7, 9].

Although many studies supported that the proper use of the ISBARQ protocol can facilitate nurses to structure clearer communication during the handover process, others reported that it was not the only factor that determined the quality of handover. In fact, the quality of handover communication also highly depends on nurses’ perceptions of their handover practice [28, 29] and their degree of understanding of the patient care plan [11]. The path coefficient in our study revealed that the understanding of the patient care plan plays a significant positive role (.206, *p* < .01) in enhancing the quality of handover, which was in line with Slade et al.’s [28, 29] and Pun et al.’s [11] studies. Thus, nurses should be encouraged to check for patients’ information regarding both diagnosis and treatment as frequently as possible to improve their understanding.

With reference to Ginsburg [23], the inadequate opportunities of asking questions and clarifying information are one of the major reasons leading to imprecise handovers. As illustrated in our findings, having the opportunity to ask questions and receiving updated information during handover respectively formed an indirect positive correlation with the quality of handover given the enablement of greater and detailed knowledge about a patient’s condition and care plan. Thus, the keys to quality handover practice are to ensure the incoming nursing staff understand the patient care plan and that the outgoing nursing staff provide them the opportunity to ask questions, as well as giving updated patient information during handover communication [24, 35]. Furthermore, our findings showed that the perception of the ISBAR protocol was not the only factor that determined the quality of handover, as indicated by the lack of a statistically significant path between these variables. In contrast to the previous research [11, 17, 28, 29], we found that the nursing staff’s positive perception of the ISBAR protocol (.010, *p* > .05) was not likely to be a motivating factor for constructive behaviour, in which the perception of ISBAR promoted a clear understanding of patient care plan among nurses merely to a small extent. Rather, the two other mediating factors, namely updated information and opportunity to ask questions, were found to pose a significant impact on their understanding of patient care plan, in turn, facilitate nurses’ self-perceived quality of handover.

### Limitations

The current study had a few limitations. To begin with, participants were recruited from a single hospital in Hong Kong; therefore, generalisation of our research findings should be made cautiously. Second, as this research only included limited number of variables, it provided only a fragment of the picture in terms of the quality of handover, more potential factors that may lead to successful quality of handover should be explored in further study. Third, due to the concern about balancing nursing staff’s heavy workload and participation in research, there was only a one-off workshop provided by the researcher. Therefore, to enhance the effect of training, a longitudinal study with multiple workshops is necessary for addressing the potential bias of the design of current research.

## Conclusion

The path analysis revealed nurses’ perception of the ISBAR protocol was not significantly correlated with the quality of handover. In our findings, we showed that nurses who expressed that they had the opportunity to ask questions self-reported a more satisfactory handover practice. In addition, nurses who obtained updated information were found to ask more questions and self-perceived a better understanding of the patient care plan at handover. Although all nurses followed the same ISBARQ protocol, they exhibited different perceptions towards handover, which resulted in different styles of communication and understanding of the patient care plan among the nurses.

Although the ISBAR protocol is considered as a useful tool that guides nurses to structure clearer handover communication, which could in turn help improve the quality of handover practice. Our findings suggested that nurses’ perception of ISBAR was not an essential predictive factor for the quality of handover. Instead, the quality of handover practices depends on the degree of nurses’ understanding of the patient care plan. Further, as illustrated in the hypothesised model, to obtain a complete understanding of the patient care plan, providing the opportunity to ask as many as questions as required to have updated information about a patient’s condition is highly regarded to enhance the quality of handover practice among nurses.

## Supplementary Information

**Additional file 1.**

## Data Availability

The datasets used and/or analysed during the current study are available from the corresponding author on reasonable request.

## Abbreviations

ISBAR: Introduction, Situation, Background, Assessment
ISBARQ: Introduction, Situation, Background, Assessment, Recommendation and Question and answer
NHPQ: Nurses Handover Perceptions Questionnaire
RMSEA: Root mean square error of approximation
CFI: Comparative fit index
TLI: Tucker–Lewis index
