# Supplementary material for: Factors associated with nurses’ perceptions, their communication skills and the quality of clinical handover in the Hong Kong context
Source: BMC Nurs. 2021 Jun 11;20:95. doi: 10.1186/s12912-021-00624-0 (PMC8196458; doi:10.1186/s12912-021-00624-0)
Supplement: Supplementary file 1 — Additional file 1. [file 12912_2021_624_MOESM1_ESM.docx]

**Title page:**

Title: Factors associated with nurses’ perceptions, their communication skills and the quality of clinical handover in a bilingual context

Author:

Jack PUN^1^

Affiliations:

1- The City University of Hong Kong, Department of English, Hong Kong SAR, China

Corresponding author: Jack Pun (email: [jack.pun@cityu.edu.hk](mailto:jack.pun@cityu.edu.hk))

Address: Department of English, 8/F, Creative Media Centre, The City University of Hong Kong, Kowloon Tong, Hong Kong SAR, China.

**Nursing Handover Perspectives Questionnaire (NHPQ)**

To participants:

This questionnaire is designed to explore the nurses’ perceptions on their handover practices in Hong Kong.

Please try to respond to all questions. All responses will be treated as anonymous and completely confidential. The information collected from this questionnaire is available to the researcher for ***research purposes only***, but not to your colleagues.

The estimated time for completing this questionnaire is 15 minutes.

If you have any questions or concerns about this study, please contact

Dr Jack Pun

Principle Investigator

Assistant Professor

Department of English, City University of Hong Kong

Email: [jack.pun@cityu.edu.hk](mailto:jack.pun@cityu.edu.hk)

Tel: 3442 9415

Thank you for your time and help.

**Section 1: Handover practices:**

Based on your own handover experience in Hong Kong, please **circle** the most appropriate option that reflects your communication practice.

|  | Items | Strongly Disagree | Disagree | Agree | Strongly Agree |
| --- | --- | --- | --- | --- | --- |
|  | I have been provided with adequate information about patients in my care. | 1 | 2 | 3 | 4 |
|  | The handover information was presented in a systematic and organised way. | 1 | 2 | 3 | 4 |
|  | I feel that important information was not given to me. | 1 | 2 | 3 | 4 |
|  | The information I received during handover is often not relevant to my patient care | 1 | 2 | 3 | 4 |
|  | The charts were available during handover to clarify information provided to me. | 1 | 2 | 3 | 4 |
|  | I have used the charts to review patient care during handover, for example drug chart, vital signs, patient allergy, FBC. | 1 | 2 | 3 | 4 |
|  | The way in which information was provided to me was easy to follow. | 1 | 2 | 3 | 4 |
|  | I was unable to keep my mind focused during handover due to excessive noise. | 1 | 2 | 3 | 4 |
|  | I think effective communication skills should be used for handover, for example clear speech, not too fast. | 1 | 2 | 3 | 4 |
|  | From my experience, handover was often interrupted by patients, their significant others or other staff. | 1 | 2 | 3 | 4 |
|  | The information I received was up to date. | 1 | 2 | 3 | 4 |
|  | I had to seek further information about my patient(s) from a nurse or nurse-in-charge after the handover. | 1 | 2 | 3 | 4 |
|  | I had the opportunity to ask questions about things I did not understand during handover. | 1 | 2 | 3 | 4 |
|  | I was asked to clarify if I have any questions about the information received. | 1 | 2 | 3 | 4 |
|  | As a result of handover, I have a clear understanding of the plan (diagnosis, treatment, discharge) for the patient(s). | 1 | 2 | 3 | 4 |
|  | I received adequate information about nursing care during handover, for examples mobility, nutrition/hydration, pain. | 1 | 2 | 3 | 4 |
|  | From my observations, important vital sign measurements are often omitted from nursing handover, for example BP **<**100, oxygen saturation **<**93%. | 1 | 2 | 3 | 4 |
|  | From my observations, important information about medication is often not given during handover, for example withheld, allergy, unavailable. | 1 | 2 | 3 | 4 |
|  | I believe using ISBAR will help me to improve communication skills with my co-workers. | 1 | 2 | 3 | 4 |
|  | I believe using ISBAR will increase patient quality and safety care. | 1 | 2 | 3 | 4 |
|  | I think ISBAR is time consuming. | 1 | 2 | 3 | 4 |
|  | I think ISBAR is not easy to implement in my handovers. | 1 | 2 | 3 | 4 |

**Section 2: Demographic Information**

1. What is your gender?

| **1** = Female | **2** = Male |
| --- | --- |

2. What is your age group? (Please circle to the number corresponding to your age group)

| 20-24  **1** | 25-29  **2** | 30-34  **3** | 35-39  **4** | 40-44  **5** |
| --- | --- | --- | --- | --- |
| 45-49  **6** | 50-54  **7** | 55-59  **8** | 60 or above  **9** |  |

3. What is your highest education level?

| **1** = Diploma | **2** = Bachelor’s Degree |
| --- | --- |
| **3** = Master and above | **4** = Other: _________________  (please specify) |

4. What is your staff position in the hospital?

| **1** = Nurse Consultant/ Ward Manager | **2** = Advanced Practice Nurse |
| --- | --- |
| **3** = Registered Nurse | **4** = Other: _________________  (please specify) |

5. How long have you been working in this current hospital/clinical site?

| **1** = 0 to 1 year | **2** = 2 to 5 years |
| --- | --- |
| **3** = 6 to 10 years | **4** = More than 10 years |

6.. How many years experiences of working in other hospital(s)/clinical site(s) do you have?

| **1** = 0 to 1 year | **2** = 2 to 5 years |
| --- | --- |
| **3** = 6 to 10 years | **4** = More than 10 years |

7. Have you ever received any professional training about communication?

If yes, please specify what kind of training was it?

| **1** = No | **2** = Yes:  (please specify)  _______________________________________________________________ |
| --- | --- |

--Thank you for your time and patience in completing this questionnaire.
